# Influence of Arbuscular Mycorrhizae on Biomass Production and Nitrogen Fixation of Berseem Clover Plants Subjected to Water Stress

**DOI:** 10.1371/journal.pone.0090738

**Published:** 2014-03-03

**Authors:** Sergio Saia, Gaetano Amato, Alfonso Salvatore Frenda, Dario Giambalvo, Paolo Ruisi

**Affiliations:** Dipartimento di Scienze Agrarie e Forestali, Università degli Studi di Palermo, Palermo, Italy; RIKEN Center for Sustainable Resource Science, Japan

## Abstract

Several studies, performed mainly in pots, have shown that arbuscular mycorrhizal symbiosis can mitigate the negative effects of water stress on plant growth. No information is available about the effects of arbuscular mycorrhizal symbiosis on berseem clover growth and nitrogen (N) fixation under conditions of water shortage. A field experiment was conducted in a hilly area of inner Sicily, Italy, to determine whether symbiosis with AM fungi can mitigate the detrimental effects of drought stress (which in the Mediterranean often occurs during the late period of the growing season) on forage yield and symbiotic N_2_ fixation of berseem clover. Soil was either left under water stress (i.e., rain-fed conditions) or the crop was well-watered. Mycorrhization treatments consisted of inoculation of berseem clover seeds with arbuscular mycorrhizal spores or suppression of arbuscular mycorrhizal symbiosis by means of fungicide treatments. Nitrogen biological fixation was assessed using the ^15^N-isotope dilution technique. Arbuscular mycorrhizal symbiosis was able to mitigate the negative effect of water stress on berseem clover grown in a typical semiarid Mediterranean environment. In fact, under water stress conditions, arbuscular mycorrhizal symbiosis resulted in increases in total biomass, N content, and N fixation, whereas no effect of crop mycorrhization was observed in the well-watered treatment.

## Introduction

Arbuscular mycorrhizal (AM) fungi are commonly occurring fungi that live in an obligate symbiotic status with the majority of land plants. AM symbiosis has a positive influence on plant growth, which is mainly attributable to the ability of AM fungi to take up nutrients from the soil — especially phosphorus [1], [2] and to a lesser extent nitrogen (N) [3], [4] — and deliver them to the roots of its host, and also to enhance the health of its host by protecting it from pathogens, pests, and parasitic plants [5]. In addition, in legume crops, AM symbiosis can improve the degree of dependence of the legume on atmospheric N_2_ [6] by increasing the uptake of key nutrients to simultaneously feed plants and nodules or through direct effects on the functioning of the legume-*Rhizobium* symbiosis [7]. Moreover, AM symbiosis can mitigate the negative effects of water stress (WS) on plant growth [8], although the underlying mechanisms of this are still unclear [9]. Nevertheless, an extensive review by Augé [10] covering hundreds of studies (which were performed mainly in pots) highlights the fact that mycorrhizal effects on plant-water relationships are often subtle, transient, and probably circumstance- and symbiont-specific.

Berseem clover (*Trifolium alexandrinum* L.) is a drought-tolerant forage legume that is well adapted to semiarid conditions and widely cultivated under rain-fed conditions in the Mediterranean, central Asia, and, most recently, the United States [11]. In Mediterranean areas, it is usually sown in the autumn and grown until early summer as a multicut forage species, thanks to its ability to regrow after cutting [12]. However, during the spring, plant regrowth is often limited by water deficiency, which negatively affects photosynthesis and symbiotic N_2_ fixation and leads to a reduction in accumulation and quality of biomass. In berseem clover, experiments performed under controlled conditions have shown that AM symbiosis can play an important role in protecting the host plant from both biotic and abiotic stresses [13], [14]. Pellegrino et al. [15], in a study that included both pot and field trials, found that the use of AM fungal inocula may be very effective in improving berseem clover productivity and quality and that the choice of the most appropriate AM fungal inoculum is essential to the success of inoculation practices. Moreover, other studies have found that AM symbiosis plays a role in determining the N_2_ fixation activity of berseem clover under low soil phosphorus concentration, at least during the early stages of the *Rhizobium*-legume interaction [16]. However, to our knowledge, no information is available about the effects of AM symbiosis on berseem growth and N_2_ fixation under water shortage conditions. Thus, we performed a field experiment to determine whether symbiosis with AM fungi can mitigate the detrimental effects of drought stress (which in Mediterranean environments often occurs during the late period of the growing season) on forage yield, quality, and symbiotic N_2_ fixation of berseem clover.

## Materials and Methods

### Ethics Statement

No specific permits were required for the described field study. The location is not protected in any way.

The experiment did not involve endangered or protected species.

### Experimental site

The trial was conducted during the 2007–2008 growing season at the Pietranera farm, which is located about 30 km north of Agrigento, Sicily, Italy (37°30’ N, 13°31’ E; 178 m asl), on a deep, well-structured, clay soil classified as a Vertic Haploxerept. Soil characteristics are presented in table S1. The climate of the experimental site is semiarid Mediterranean, with a mean annual rainfall of 552 mm, mostly in the autumn and winter (74%) and in the spring (18%). The dry period lasts from May to September. The mean air temperature is 15.9°C in autumn, 9.8°C in winter, and 16.5°C in spring. The average minimum and maximum annual temperatures are 10.0°C and 23.3°C, respectively. The weather data were collected from a weather station located within 500 m of the experimental site.

**Figure 1:**
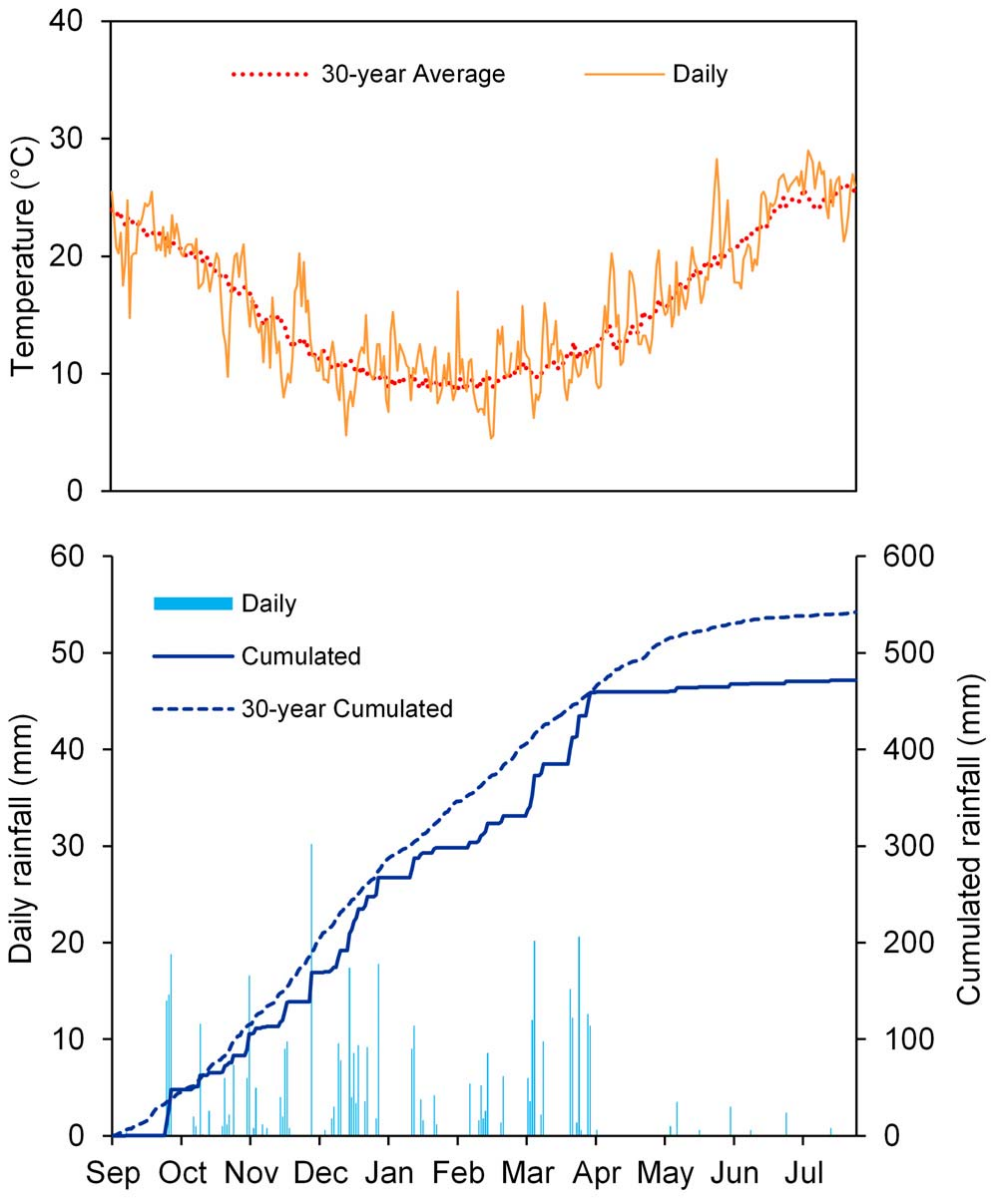
Cumulative daily rainfall and mean air temperatures at the experimental site during the 2007–2008 growing season. 30-year (1980–2010) average cumulative daily rainfall and temperatures are included.

During the 2007–2008 growing season (from September to May), total rainfall was 516 mm (slightly lower than the long-term average) and it was well distributed during fall and winter (Fig. 1). Total rainfall during the spring (91 mm) was markedly lower than normal (–30%). Average, minimum, and maximum temperatures were similar to long-term averages.

### Experimental design and crop management

A split-plot design with four replicates was adopted, with plot sizes of 36 m^2^ each (12 rows 12 m long and 0.25 m apart). The main plot was soil water availability: crops were subjected to WS or well-watered (WW) conditions. The subplot was mycorrhization: inoculation of berseem clover seeds with AM spores (+AM) or suppression of AM symbiosis by means of fungicide treatments (–AM). In WS treatment, berseem clover was grown entirely under rain-fed conditions, whereas in WW three sprinkle irrigations, 30 mm each, were done by irrigating when soil water dropped below a value of about 0.7 of the available soil water capacity (116, 125, and 132 days after sowing, DAS). The amount of water applied at each irrigation point was sufficient to bring soil water content to field capacity. Soil moisture content was measured gravimetrically (in the 0–0.40-m layer) once per month from sowing to 110 DAS and every 3 days thereafter.

Natural AM spore population in the field was measured before sowing by the wet-sieving method [17] and consisted of a mycorrhizal consortium formed mainly by *Glomus*-group AM species and *Acaulospora*. Overall AM spore density was 5 spores per 10 g air-dried soil. AM hyphal mass in the soil was not detected. In –AM treatment, suppression of the natural AM symbiosis was achieved by spraying the plots/plants with systemic fungicides: Captan (20 mg a.i. m^–2^), Carbendazim (20 mg a.i. m^–2^), and Benomyl (20 mg a.i. m^–2^) were used at sowing time to suppress AM spore germination; then, Benomyl, Fenpropimorph, and Carbendazim were applied once per month during the growth of the crop, at rates of 10, 5, and 5 mg a.i. m^–2^, respectively, to reduce AM hyphal growth and symbiotic activity. These fungicides have no toxic effects on plant growth [18], [19]. Inoculation with AM fungi involved the application of a commercial AM inoculum at a rate of 12 g kg^–1^ of seed. The inoculum consisted of a mixture of spores of *Rhizophagus irregularis* (formerly *Glomus intraradices*) and *Funneliformis mosseae* (formerly *G. mosseae*), each of which was present at a rate of 1000 spores g^–1^ of inoculum. Berseem clover seed was not inoculated with Rhizobium before planting because prolific inoculation occurs naturally at this site.

Soil, cropped in the previous growing season with durum wheat (*Triticum durum* Desf.), was plowed to a depth of 30 cm in the summer and then shallowly harrowed twice to control weeds and prepare suitable seedbed conditions. No herbicides were applied. Berseem clover (cv Lilibeo) was sown by hand at the end of December at a rate of 1200 viable seeds m^–2^. Weeds were removed by hand during the growing season.

The entire plot was cut at a 5-cm stubble height at 76 DAS (C1) and again at 116 DAS (C2); at each cut, a subplot (2.25 m^2^) was used to determine plant hypogeic (belowground) biomass. Starting at C2, water treatment was established, and crop regrowth was monitored by cutting different subplots (2.25 m^2^) after 7 (R7), 14 (R14), 21 (R21), and 28 days (R28).

Symbiotic N_2_ fixation was assessed at R14 and R28 using the ^15^N-isotope dilution technique by applying a ^15^N-labeled fertilizer ([NH_4_]_2_SO_4_ with an isotopic composition of 10 atom% ^15^N) at 116 DAS, soon after C2. ^15^N-labeled fertilizer was applied as drench at a rate of 8 kg N ha^–1^ to the subplots assigned to R14 and R28. In all cases, the rest of the plot outside the ^15^N-labeled areas received a topdressing of ammonium sulfate in an amount equivalent to that in the subplots. Ryegrass (*Lolium multiflorum* var. *Westerwoldicum* cv Elunaria) was chosen as a reference crop because its growth rate and regrowth ability are similar to those of berseem clover [20]; ryegrass received the same treatments as berseem clover.

### Plant harvests and analyses

At C1 and C2, the removed biomass (above cutting height) was weighed, and two subsamples of 1 kg of fresh matter each were taken. The first was separated into leaves, stems, and heads, whereas the second was dried at 65°C until a constant weight was reached. Furthermore, on subplots assigned to destructive measures, both residual aboveground biomass (below cutting height) and hypogeic biomass (in the first 20 cm of soil) were measured. The residual aboveground biomass was separated into leaves and stems. All botanical fractions were weighed and dried at 65°C to a constant weight. The leaf area index (LAI) of the residual and removed leaves was immediately measured on a 10-g subsample. On subplots assigned to R7, R14, R21, and R28 cuts, total aboveground biomass (above cutting height + below cutting height) and hypogeic biomass were measured. Samples of total aboveground biomass were separated into botanical fractions, and LAI was measured using the same method described earlier. Biomass samples from R14 and R28 were analyzed for total N and ^15^N enrichment by means of an elemental analyzer–isotope ratio mass spectrometer (Carlo Erba NA1500).

AM root colonization was measured according to Giovannetti and Mosse [21] on root samples (from five plants randomly chosen from each plot) collected at 55 DAS, at R14, and at R28; roots were stained with 0.05% trypan blue in lactic acid according to Phillips and Hayman [22].

### Calculations and statistical analysis

Data on biomass ^15^N enrichment were used to calculate the percentage of N_2_ derived from symbiotic N_2_ fixation (%Ndfa) according to Fried and Middelboe [23]:



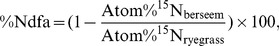



where Atom%^15^N_berseem_ represents the Atom%^15^N excess of berseem tissues and Atom%^15^N_ryegrass_ represents the Atom%^15^N excess of ryegrass tissue. The ^15^N-natural abundance of the atmosphere (0.3663%^15^N) was used to calculate the Atom%^15^N excess of both crops. The amount of N fixed by berseem clover was estimated as:



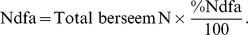



Analysis of variance [24] was performed separately for each cut according to the experimental design. All measured variables were assumed to be normally distributed. All variables corresponding to proportions were arcsine transformed before analysis to ensure a better fit with the Gaussian law distribution.

## Results

Soil moisture did not drop below a value of about 0.7 of the available soil water capacity until C2. As expected, from C2 to R28, soil moisture content was always significantly higher in WW than WS conditions (25.4% vs. 19.4% across all sampling dates; P < 0.001), and the differences between the two treatments increased with time. Soil moisture content was not affected by crop mycorrhization, and the interaction with soil moisture regimen was never significant.

At 55 DAS, root colonization by AM fungi was significantly lower in the –AM crops than in the +AM crops (7.0% and 32.4%, respectively; P < 0.001). At C1, both total and residual biomass of berseem clover, and LAI of total biomass and of residual biomass, were significantly higher in the +AM than the –AM crops (Table 1). Also, root biomass (up to 20 cm depth) was significantly higher in +AM than in –AM. At C2, no significant effect of mycorrhization was observed on biomass accumulation, canopy traits (LAI and percentage of leaves), or root biomass.

**Table 1:** Field response of shoots and roots of berseem clover to AM inoculation at first cut (C1, at 76 days after sowing - DAS) and second cut (C2, at 116 DAS).

		C1 (76 DAS)			C2 (116 DAS)		
		–AM	+AM		–AM	+AM	
Total aboveground biomass	g m^–2^	159	187	**	548	568	ns
Removed (Rem)	g m^–2^	113	136	**	393	410	ns
Residual (Res)	g m^–2^	46	51	*	155	158	ns
Leaves in Rem	% on Rem biomass	74.7	68.2	**	40.6	41.5	ns
Leaves in Res	% on Res biomass	32.2	32.8	ns	28.6	28.5	ns
Total LAI	m^2^ m^–2^	2.96	3.39	*	5.93	6.30	ns
LAI of Res	m^2^ m^–2^	0.44	0.51	*	1.31	1.33	ns
Root biomass	g m^–2^	35	41	*	36	36	ns

+AM, inoculation with arbuscular mycorrhizal spores; –AM, suppression of arbuscular mycorrhizal symbiosis.

* and ** indicate significant differences at 0.05 and 0.01 probability level, respectively; ns indicates differences not significant.

**Figure 2:**
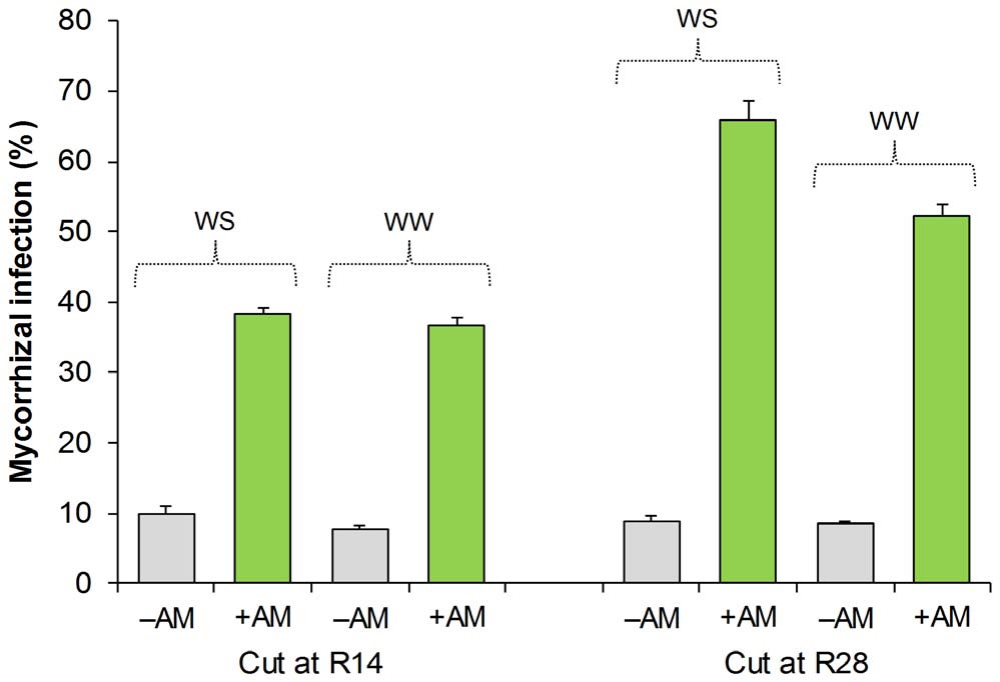
Mycorrhizal infection of berseem clover roots as affected by water availability and mycorrhization treatment. Data are means (n = 4) ± S.E. measured at R14 (14 days after C2) and R28 (28 days after C2). WW, well-watered; WS, water-stressed; +AM, inoculation with arbuscular mycorrhizal spores; –AM, suppression of arbuscular mycorrhizal symbiosis.

Tests of significance for the effect of water treatment and crop mycorrhization on the traits measured during crop regrowth (at R7, R14, R21, and R28) are reported in Table 2. At R14 (14 days after the establishment of water treatment), AM infection was, on average, 8.8% in –AM and 37.7% in +AM (significantly different at P < 0.001); at this time, no effect of the soil moisture regime was observed on root colonization by AM fungi (Fig 2). At R28, root colonization by AM fungi was very low in the –AM treatment areas, irrespective of soil moisture regime (average of 8.7%), whereas in +AM treatment areas, root colonization was significantly higher in the WS areas than in the WW areas (66.0% and 52.4%, respectively).

**Table 2:** F values and significance levels for water availability (W) and crop mycorrhization (M) on measured traits of regrowth, conducted separately for each sampling date (R7, R14, R21, and R28).

Treatments		Biomass regrowth	Taproot biomass	LAI	Stems %	Heads %	Above ground N		Taproot N		Ndfa		Mycorrhizal infection
		g m^–2^	g m^–2^		on biomass regrowth		g kg^–1^	g m^–2^	g kg^–1^	g m^–2^	%	g m^–2^	%
Water availabilty (W)													
R7		22.54*	0.14^ns^	15.30*	0.13^ns^	†	-	-	-	-	-	-	-
R14		20.03*	8.96^ns^	262.25***	3.19^ns^	33.14*	0.27^ns^	25.67*	4.61^ns^	50.03**	7.83^ns^	57.77**	2.52^ns^
R21		110.99**	2.63^ns^	48.46**	38.79**	51.06**	-	-	-	-	-	-	-
R28		10.42*	0.03^ns^	92.74**	233.98***	246.77***	3.81^ns^	81.93**	0.06^ns^	0.04^ns^	16.14*	79.73**	19.87*
Crop mycorrhization (M)													
R7		0.08^ns^	0.47^ns^	3.42^ns^	2.05^ns^	†	-	-	-	-	-	-	-
R14		1.00^ns^	2.82^ns^	0.49^ns^	0.13^ns^	0.16^ns^	0.03^ns^	1.80^ns^	0.47^ns^	2.80^ns^	0.00^ns^	0.63^ns^	1348.18***
R21		22.89**	1.03^ns^	0.06^ns^	0.14^ns^	3.62^ns^	-	-	-	-	-	-	-
R28		13.08*	0.01^ns^	4.79^ns^	1.15^ns^	0.11^ns^	0.82^ns^	0.65^ns^	0.17^ns^	0.32^ns^	14.38**	21.38**	887.47***
W × M													
R7		4.93^ns^	3.14^ns^	1.38^ns^	0.23^ns^	†	-	-	-	-	-	-	-
R14		0.86^ns^	0.46^ns^	0.06^ns^	5.71^ns^	0.16^ns^	0.43^ns^	2.20^ns^	0.00^ns^	0.38^ns^	1.05^ns^	4.74^ns^	0.03^ns^
R21		6.22*	0.01^ns^	4.99^ns^	0.73^ns^	4.66^ns^	-	-	-	-	-	-	-
R28		29.78**	1.49^ns^	0.41^ns^	0.01^ns^	4.31^ns^	0.04^ns^	7.33*	0.00^ns^	3.41^ns^	6.68*	21.55**	14.88**

*, ** and *** indicate significant differences at 0.05, 0.01 and 0.001 probability level, respectively; ^ns^ indicates differences non significant.

† Heads were absent in all the treatments.

-, not detected.

**Figure 3:**
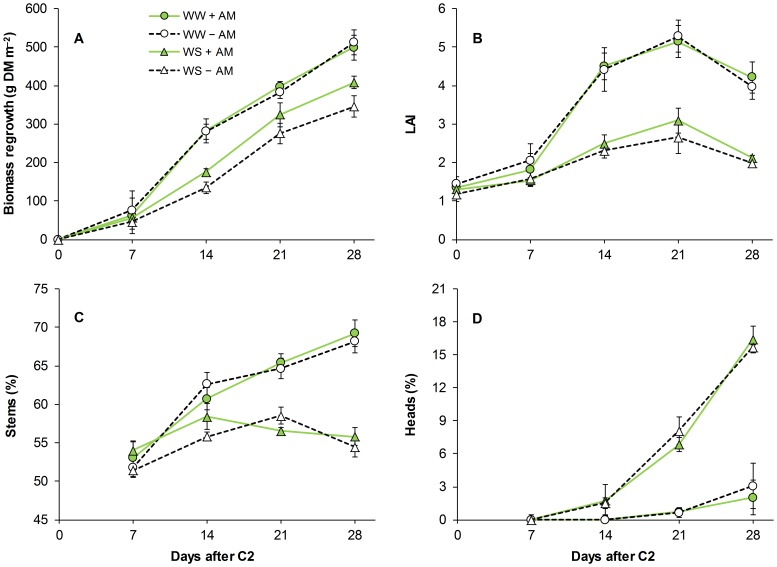
Biomass regrowth (A), LAI (B), and incidence of stems (C) and heads (D) as a percentage of berseem clover biomass as affected by water availability and mycorrhization treatment. Data are means (n = 4) ± S.E. measured after 7, 14, 21 and 28 days from second cut (C2). WW, well-watered; WS, water-stressed; +AM, inoculation with arbuscular mycorrhizal spores; –AM, suppression of arbuscular mycorrhizal symbiosis.

The increase in water availability in WW compared to WS led to significant increases in biomass regrowth starting at R7 (Table 2 and Fig. 3A). The differences between WW and WS increased until R14 and then remained constant until R28. Crop mycorrhization increased biomass regrowth in WS but not in WW and the difference between +AM and –AM increased with time.

Irrigation led to an increase in LAI and stem percentage and a decrease in head percentage with differences between WW and WS increasing with time (Fig. 3B, 3C and 3D). Crop mycorrhization did not affect LAI or the incidence of stems and heads as a percentage of crop biomass in either WS or WW (Table 2 and Fig. 3B, 3C and 3D).

Both water treatment and crop mycorrhization almost never affected taproot biomass (38.4 g m^-2^ averaged across all sampling dates, with slight increases with time).

**Figure 4:**
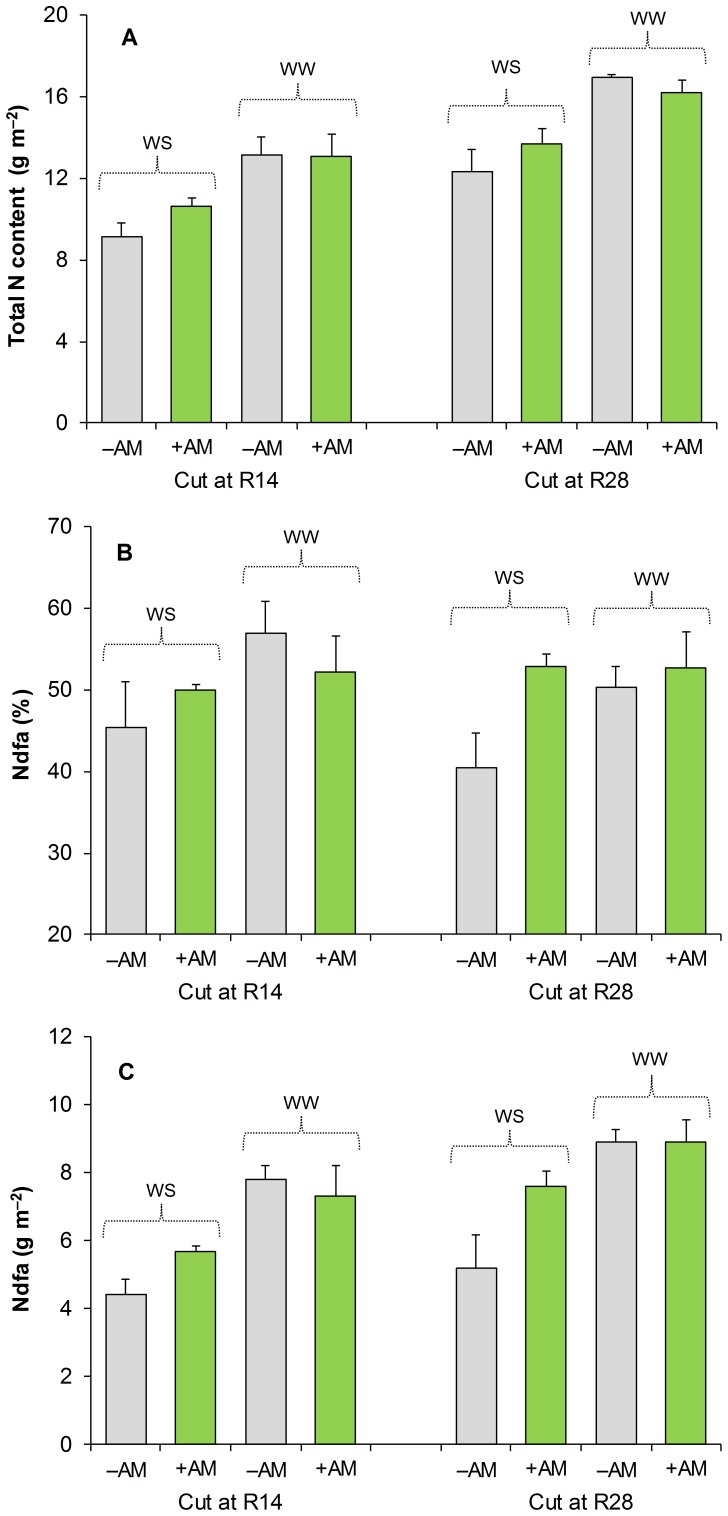
Total N content (A), Nitrogen derived from the atmosphere as a percentage of total N content (B) and total fixed N (C) of berseem clover as affected by water availability and mycorrhization treatment. Data are means (n = 4) ± S.E. measured at R14 (14 days after C2) and R28 (28 days after C2). WW, well-watered; WS, water-stressed; +AM, inoculation with arbuscular mycorrhizal spores; –AM, suppression of arbuscular mycorrhizal symbiosis.

N concentration did not vary according to the treatment applied (on average 30.5 g kg^-1^ and 24.7 g kg^-1^ in aboveground biomass at R14 and R28, respectively, and 18.6 g kg^-1^ and 17.6 g kg^-1^ in taproot biomass at R14 and R28, respectively). Irrigation increased total N content both at R14 and R28 (Fig. 4A). Crop mycorrhization resulted in an increase in total N content in WS but not in WW.

Irrigation increased total N fixed both at R14 (on average 50% compared to WS) and at R28 (on average 39% compared to WS) (Fig. 4C). At R14, crop mycorrhization did not affect Ndfa (as both a percentage of total N content and the amount of N fixed on an area basis). On the contrary, at R28, Ndfa values were significantly higher in +AM than in –AM in the WS treatment, whereas no effects of crop mycorrhization were observed in the WW treatment.

## Discussion

On the whole, when berseem clover did not experience WS (i.e., in WW treatments), aboveground biomass production was about 12 Mg dry matter ha^–1^; this is similar to the biomass obtained in the same environment when rainfall during the growing season was 30% higher than the long-term average [12], [20]. On average, no differences between –AM and +AM conditions were observed in terms of total aboveground biomass production (11.7 vs. 12.0 Mg dry matter ha^–1^, calculated as a sum of C1+C2+R28 in WW treatments). This result is in contrast with the findings of Pellegrino et al. [15], who observed, in a field trial, an increase of berseem clover productivity (from 6.5 to 10.0 Mg dry matter ha^–1^) following inoculation with AM fungi in comparison with a non-inoculated control. Because the main recognized plant benefit from AM symbiosis consists of improved access to limited soil resources (i.e., nutrients—especially phosphorus—and water), it is probable that, in our experiment, in contrast to that of Pellegrino et al. [15], the high level of soil fertility and the adequate water availability made mycorrhizal symbiosis superfluous. In such conditions, the only advantage of AM symbiosis that we observed occurred at the first cut (C1, at 76 DAS), at which time mycorrhiza inoculation resulted in increased root and shoot dry matter. According to Miller [25], during the early phase of the crop cycle, when roots are not yet well developed, AM symbiosis can make an important contribution to plant growth by enhancing nutrient acquisition.

In contrast to the WW conditions, under WS conditions, the AM symbiosis enhanced aboveground biomass production of berseem clover. This result is consistent with the findings of other authors who found, in pot-based studies, a beneficial effect of AM symbiosis under water restriction for both legumes [26], [27] and non-legume plants [28], [29]. The AM fungi contribution to plant drought tolerance is well documented (as reviewed by Augé [10]), but the underlying mechanisms are still unclear. These include hyphal water uptake [28], enhanced nutrient uptake —especially phosphorus— and enhanced plant osmotic adjustment [30], [31], which favor plant water uptake from soil.

Numerous pot-based experiments have clearly indicated that AM symbiosis can greatly assist nodulation and N_2_ fixation of legumes [32], [33]. We observed significant increases in Ndfa (as both percentage of total N content and amount of N fixed on an area basis) only under WS conditions. This result may be attributable to the enhancement of nutrient uptake offered by AM fungi under WS conditions, which allowed the plants to satisfy both the nodule’s and their own nutritional needs for growth. In this context, as is well known, an increase in phosphorus acquisition can play an important role in stimulating both nodulation and N_2_ fixation [34]. Our research was performed in soil that is very rich in phosphorus; such conditions can strongly reduce the mycorrhizal benefit in terms of plant growth and phosphorus uptake [35]. However, in some cases, under WS conditions, AM plants are able to absorb more phosphorus than non-mycorrhizal plants, even when this element is highly available in the soil [9]; in fact, phosphorus effectively becomes less available with decreasing soil moisture [36], [37], so that AM colonization and effective development of external mycelium can be extremely important for nutrient uptake in these situations. Moreover, other authors [27] highlighted that the advantages of the AM symbiosis in WS conditions are related to the defense of plant roots against the oxidative damage generated by drought, which can then ensure a certain level of protection against nodule senescence (with positive effects on the N_2_ fixation process).

In WS treatments, the benefits of AM symbiosis were effective only at R28 (i.e., at 28 days after C2), whereas no differences were observed at R14 (i.e., 14 days after C2). This result can be explained by considering that, in legume forage crops subjected to cuts, regrowth ability is highly dependent on the plant’s capacity to mobilize carbon and N reserves stored in roots and stubble and on photosynthetic activity of the residual leaf area [12], [38], [39]. Thus, during the early regrowth phase (from C2 to R14), carbon can represent a limiting factor, and in our experiment, it is likely that the high sink strength of the growing shoots limited the availability of resources for the AM fungus, counterbalancing the potential benefit offered by AM symbiosis; in the late regrowth phase (from R14 to R28), the photosynthetic apparatus reached an extent so great that plants were able to guarantee sufficient availability of the photosynthates to sustain both AM symbiosis and plant growth. The lower percentage of root colonization by AM fungi observed in R14 with respect to R28 seems to confirm the plant’s difficulty in sustaining mycorrhizal symbionts during the early regrowth phase.

In conclusion, the results of our study confirm that, in field conditions, AM symbiosis can play an important role in the growth of plants under conditions of abiotic stresses such as WS. In fact, AM symbiosis in berseem clover subjected to WS mitigated the negative effects of drought on plant growth also through the stimulation of N_2_ fixation. Moreover, AM symbiosis made an important contribution during the early growth phase, when nutrient uptake is limited by the relatively poorly developed plant root system.

## Supporting Information

Table S1 Physical and chemical characteristics of the top layer (0–0.40-m) of soil at the experimental site.
(DOCX)
