# Supplementary material for: Influence of Arbuscular Mycorrhizae on Biomass Production and Nitrogen Fixation of Berseem Clover Plants Subjected to Water Stress
Source: PLoS One. 2014 Mar 3;9(3):e90738. doi: 10.1371/journal.pone.0090738 (PMC3940947; doi:10.1371/journal.pone.0090738)
Supplement: Table S1 — Physical and chemical characteristics of the top layer (0–0.40-m) of soil at the experimental site. (DOCX) [file pone.0090738.s001.docx]

Table S1. Physical and chemical characteristics of the top layer (0–0.40-m) of soil at the experimental site.

| Soil type |  | Vertic Haploxerept |
| --- | --- | --- |
| Clay | % | 49.8 |
| Silt | % | 23.2 |
| Sand | % | 27.0 |
| pH (1:2.5 H_2_O) |  | 8.0 |
| Total limestone | % | 16.0 |
| Total C (Walkley-Black) | g kg^−1^ | 17.1 |
| Total N (Kjeldahl) | g kg^−1^ | 1.24 |
| Available P_2_O_5_ (Olsen) | mg kg^−1^ | 107 |
| Total P_2_O_5_ | g kg^−1^ | 1.47 |
| CEC | cmol kg^−1^ | 31.0 |
| Water content at field capacity (pF 2.5) | cm^3^ cm^−3^ | 0.38 |
| Permanent wilting point (pF 4.5) | cm^3^ cm^−3^ | 0.16 |
